# Macrophage Phenotype and Function in Liver Disorder

**DOI:** 10.3389/fimmu.2019.03112

**Published:** 2020-01-28

**Authors:** Lang Dou, Xiaomin Shi, Xiaoshun He, Yifang Gao

**Affiliations:** ^1^Organ Transplantation Center, The First Affiliated Hospital, Sun Yat-sen University, Guangzhou, China; ^2^Guangdong Provincial Key Laboratory of Organ Donation and Transplant Immunology, The First Affiliated Hospital, Sun Yat-sen University, Guangzhou, China; ^3^Guangdong Provincial International Cooperation Base of Science and Technology (Organ Transplantation), The First Affiliated Hospital, Sun Yat-sen University, Guangzhou, China

**Keywords:** hepatic macrophages, Kupffer cells, alcoholic liver disease, hepatocellular carcinoma, viral hepatitis, non-alcoholic steatohepatitis

## Abstract

Hepatic macrophages are a remarkably heterogeneous population consisting of self-renewing tissue-resident phagocytes, termed Kupffer cells (KCs), and recruited macrophages derived from peritoneal cavity as well as the bone marrow. KCs are located in the liver sinusoid where they scavenge the microbe from the portal vein to maintain liver homeostasis. Liver injury may trigger hepatic recruitment of peritoneal macrophages and monocyte-derived macrophages. Studies describing macrophage accumulation have shown that hepatic macrophages are involved in the initiation and progression of various liver diseases. They act as tolerogenic antigen-presenting cells to inhibit T-cell activation by producing distinct sets of cytokines, chemokines, and mediators to maintain or resolve inflammation. Furthermore, by releasing regenerative growth factors, matrix metalloproteinase arginase, they promote tissue repair. Recent experiments found that KCs and recruited macrophages may play different roles in the development of liver disease. Given that hepatic macrophages are considerably plastic populations, their phenotypes and functions are likely switching along disease progression. In this review, we summarize current knowledge about the role of tissue-resident macrophages and recruited macrophages in pathogenesis of alcoholic liver disease (ALD), non-alcoholic steatohepatitis (NASH), viral hepatitis, and hepatocellular carcinoma (HCC).

## Introduction

Hepatic macrophages, consisting of Kupffer cells (KCs) and recruited macrophages, are the largest population of innate immune cells in the liver. In the healthy rodent liver, macrophages comprise around 20–25% of non-parenchymal cells (1, 2); the high occupancy implies that the hepatic macrophages play a vital role in maintaining liver function and homeostasis. KCs, self-renewing tissue-resident phagocytes, are located in the liver sinusoids. During homeostasis, distinct Fc and scavenger receptors are expressed on the KC surface, which allows them to recognize modified self-molecules, resulting in clearing of apoptotic cells, cell debris, and immune complex (3, 4). Additionally, KCs are involved in controlling the iron (5), cholesterol (6), and bilirubin (7) balance of the blood. KCs also express a wide range of pattern recognition receptors (PRRs), including toll-like receptors (TLRs) (8), nucleotide oligomerization (NOD)-like receptors (9), and retinoic acid-inducible gene I (RIG-I)-like receptors (10). These receptors assist KCs to recognize and eliminate invading foreign pathogens.

Hepatic macrophages form highly heterogeneous populations, and several markers have been used to distinguish between KCs and recruited macrophages. In mice, KCs were found to express a unique maker C-Type Lectin Domain Family 4 Member F (CLEC4F) and can be characterized as CD11b+, F4/80+, TIM4+, and CLEC4F+ cell populations (11). The bone-marrow-derived macrophages are CD11b+, F4/80+, CCR2+, and CX3CR1+. MacParland et al. showed that human hepatic macrophages could be classified as CD68+ MACRO+ KCs and CD68+ MACRO– recruited macrophages in the steady state using single-cell analysis (12). According to activation programs, hepatic macrophages can be broadly divided into classically activated pro-inflammatory and alternatively activated anti-inflammatory phenotypes (13, 14). Pro-inflammatory macrophage stimuli lipopolysaccharide (LPS) and interferon (IFN)-γ activate signal transducers and activators of transcription (STAT)1, myeloid differentiation factor 88 (MyD88), Toll-interleukin 1 receptor domain containing adaptor protein (MaL/Tirap), and IFN regulatory factor (IRF)-dependent pathways, resulting in the release of interleukin (IL)-1β, IL-6, tumor necrosis factor (TNF), reactive oxygen species (ROS), and nitric oxide synthase (14–16). These macrophages are likely to contribute to hepatic inflammation and damage in distinct liver diseases. Anti-inflammatory macrophages exhibit high phagocytic capacity and produce high levels of arginase 1 as well as IL-10 via activating Janus kinase (JAK)1 and JAK3; they are featured by immunoregulation and tissue remodeling (14, 16).

It has been suggested that hepatic macrophages have two origins (17, 18): recruited macrophages derived from the hematopoietic stem cells and tissue-resident macrophages from the yolk sac. HSC-derived macrophages differentiate from circulating myeloid precursor cells from the bone marrow; this process is mediated by colony-stimulating factor (CSF)-1 (17, 18). The majority of KCs are believed to develop from the yolk sac before the appearance of HSCs (18). However, this theory has been challenged by a recent study that revealed a common progenitor for tissue-resident macrophages, called premacrophages, which were generated early in development and had colonized the whole embryo from embryonic day 9.5. Tissue-specific sets of transcriptional regulators control the differentiation of premacrophages into tissue-resident macrophages, whereby the development of KCs is regulated by Id3, a transcription factor inhibitor of DNA binding 3, and inactivation of Id3 causes KC deficiency in adults (19).

Hepatic recruited macrophages are derived from not only circulating monocytes but also macrophages of different compartments. Circulating monocytes are classified into CD11b+Ly6C^hi^ (20) and CD11b+Ly6C^low^ (21) in mice. CD11b+Ly6C^hi^ subsets can infiltrate into the liver during inflammation (20), whereas the Ly6C^low^ monocytes serve as sentinels to scavenge microparticles and cell debris in the capillaries (21). Monocytes may downregulate Ly6C expression after infiltration and before differentiation (22). Recent findings suggest that self-reviewing peritoneal cavity macrophages, characterized by F4/80^hi^GATA6+, can rapidly migrate to the liver through the mesothelium in response to a sterile injury (23). This result suggests that the composition of hepatic macrophages may be more complicated than expected. Numerous studies have shown that hepatic macrophages are involved in the progression of inflammation and fibrosis and, therefore, hold the key to controlling the pathogenesis of liver disease (14, 15, 24). In this review, we will summarize current knowledge about hepatic macrophages in pathogenesis of alcoholic liver disease (ALD), non-alcoholic steatohepatitis (NASH), hepatitis B virus/hepatitis C virus (HBV/HCV), and hepatocellular carcinoma (HCC) with a particular focus on KCs and monocyte-derived macrophages.

## Hepatic Macrophages in ALD

Chronic alcohol consumption, the primary cause of ALD, results in a broad range of disorders, including liver steatosis, alcoholic hepatitis, chronic hepatitis, HCC, liver fibrosis, and/or cirrhosis (25–27). It has been documented that hepatic macrophages accumulate within the portal tracts of ALD patients (28), whereas the depletion of hepatic macrophages via the administration of gadolinium chloride (GdCl_3_) prevents alcohol-induced liver inflammation in the rat (29). These results suggest that hepatic macrophages play a central role in the pathogenesis of ALD.

**Figure 1:**
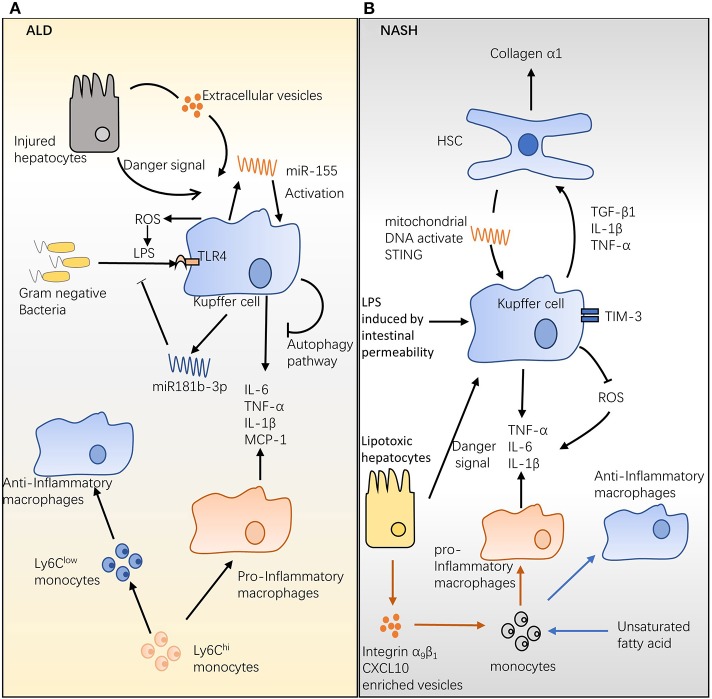
Hepatic macrophages in alcoholic liver disease (ALD) and non-alcoholic steatohepatitis (NASH). **(A)** The role of hepatic macrophages in ALD. Chronic alcohol consumption disrupts the intestinal barrier, which increases the permeability of the gut and allows Gram-negative bacteria to migrate into the portal circulation. Lipopolysaccharide (LPS) expressed on Gram-negative bacteria activates Kupffer cells (KCs) and promotes interleukin (IL)-6, tumor necrosis factor (TNF)-α, IL-1β, and monocyte chemoattractant protein (MCP)-1 release. Hepatocytes injured by alcohol consumption activate KCs via danger signal and CD40-containing extracellular vesicles. Chronic alcohol ingestion induces microRNA (miR)-155 and miR181b-3p expression; the former activates KCs and promotes inflammatory production, while the latter regulates LPS-induced inflammation. The Ly6C^hi^ monocyte can differentiate into pro-inflammatory and anti-inflammatory macrophages during ALD, and the ratio of these two populations may mediate ALD development. **(B)** The role of hepatic macrophages in NASH. High levels of LPS induced by increasing intestinal permeability and/or danger signal from lipotoxic hepatocytes stimulate KCs; activated KCs produce the survival signals, transforming growth factor β, IL-1β, and TNF-α, which stimulate hepatic stellate cells and increase generation of hepatic collagen α1, ultimately triggering fibrosis. Mitochondrial DNA from hepatocytes of high-fat diet (HFD)-fed mice activates KCs and promotes cytokine release, steatosis, and inflammation. Conversely, TIM-3 expressed on hepatic macrophages protects animals from HFD-induced NASH by inhibiting reactive oxygen species production. An HFD augments the infiltration of bone-marrow-derived monocytes into the liver and further differentiates them into protective anti-inflammatory macrophages.

One hypothesis for this effect is that ethanol ingestion disrupts the intestinal barrier, which increases the permeability of the gut, thereby enhancing the migration of Gram-negative bacteria into the portal circulation (30, 31) and leading to ALD pathogenesis. The ligation of LPS with the CD14/TLR4 receptor complex on KCs triggers the downstream IL-1 receptor-associated kinase (IRAK) and inhibitor of nuclear factor kappa-B kinase (IKK) pathways, resulting in the release of the inflammatory cytokines IL-6 and TNF-α and chemokines, such as monocyte chemoattractant protein (MCP-1) (32) (Figure 1A). These mediators augment inflammation and alcohol-induced liver injury in ALD (32). Compared with wild-type (WT) mice, alcohol-fed mice are more sensitive to LPS and produce more MCP-1 (33) and TNF-α (34) post stimulation. Recent studies showed that a small non-coding RNA, termed microRNA (miRNA), is involved in regulating macrophage infiltration, activation, and ALD progression (Figure 1A). Unbiased analysis of miRNA revealed that miR181b-3p released by KCs regulated TLR4 signaling during ethanol consumption (35). In ethanol-fed rats, the overexpression of miR181b-3p inhibited importin α5 expression and suppressed LPS-induced TNF-α expression in KCs (35). In a study in mice, chronic alcohol feeding promoted miR-155 production by KCs via the nuclear factor kappa-light-chain-enhancer of activated B cells (NF-κB) pathway (36). A later study revealed that macrophage infiltration induced by chronic alcohol consumption was reduced in miR-155-deficient mice (37). In the same study, knockout of miR-155 also alleviated the inflammation and steatosis triggered by chronic alcohol ingestion (37).

During ALD, hepatocytes injured by alcohol consumption can activate KCs. Acute and chronic ethanol exposure stimulates KCs via danger-associated molecular patterns produced by injured hepatocytes (38) (Figure 1A). Additionally, Verma et al. found that ethanol exposure stimulated hepatocytes to produce considerably more CD40L-containing extracellular vesicles in a caspase-3-dependent manner, ultimately triggering macrophage activation and production of MCP-1, TNF-α, and ROS (39) (Figure 1A). Genetic knockout of CD40 (CD40-/-) or the caspase-activating TNF-related apoptosis-inducing ligand (TRAIL) receptor (TR-/-) protected mice from alcohol-induced injury (39). Notably, during alcohol exposure, KCs are a major source of ROS, which is essential for LPS sensitization (40) and inflammatory cytokine production (41) (Figure 1A). In a chronic-plus-binge ethanol-feeding model, KCs show extracellular signal-regulated kinase 1/2 (ERK1/2) signaling attenuation and TNF-α production impairment, when they are pretreated with ROS generation inhibitor NADPH oxidase (40, 41). It has been documented that the cannabinoid receptor 2 (CB2) expressed on KCs protects mice from ALD via an autophagy pathway (Figure 1A). This effect is supported by the findings that mice with specifically targeted deletion of the CB2 receptor (CB2^Mye−/−^) or autophagy gene ATG5 (ATG5^Mye−/−^) had exacerbated liver inflammation and alcohol-induced steatosis (42). Upon exposure to LPS, KCs isolated from CB2^Mye−/−^ mice showed a pro-inflammatory phenotype that is characterized by an increased expression of chemokines IL-1β, IL-1α, IL-6, TNF-α, and CCL3 (42). These data suggest that KCs are activated toward a pro-inflammatory phenotype that increases liver inflammation and damage during ALD.

The role of recruited macrophages in ALD is less well studied. Chronic alcohol administration increases the population of recruited macrophages in the mouse liver (43). In an animal model, ethanol feeding promoted the differentiation of Ly6C^hi^ monocytes into tissue-damaging pro-inflammatory macrophages (43). Moreover, phagocytosis of apoptotic hepatocytes allows Ly6C^hi^ monocytes/macrophages to switch to Ly6C^low^ monocytes/macrophages, which then differentiate into tissue-protective macrophages (43) (Figure 1A). It has been suggested that the ratio of these two subsets determines the role of recruited macrophages in the pathogenesis of ALD (43).

## Hepatic Macrophages Contribute to Nash

About 20% of patients who suffer from non-alcoholic fatty liver disease will develop NASH, which is defined by the existence of progressive fibrosis and steatosis with inflammation, ultimately leading to HCC and cirrhosis. To date, the pathogenesis of NASH is still obscure, but several risk factors are known to be involved in the process, ranging from oxidative stress, insulin resistance, cytokines, and epigenetic modification to microbiota alteration and environmental elements (44).

One connection between KCs and NASH is the presence of hepatic stellate cells (HSCs). NASH augments endotoxin influx by increasing intestinal permeability; the high level of endotoxin and/or danger signal from lipotoxic hepatocytes can stimulate KCs (45) (Figure 1B). Activated KCs produce transforming growth factor (TGF)-β1, which stimulates HSCs and increases the generation of hepatic collagen-α1(I), eventually triggering fibrosis (46) (Figure 1B). In comparison with that in controls, collagen-α1(I) messenger RNA (mRNA) was substantially increased in carbon tetrachloride (CCl_4_)-treated mice, and this increase was abolished in TGF-β1-knockout mice (47). In addition, IL-1β and TNF-α production by stimulated KCs was required to maintain HSC survival via the NF-κB pathway (48) (Figure 1B). In a low-serum media model, hepatic macrophages protected HSCs from apoptosis, and, in the same model, neutralization of IL-1 and TNF inhibited the protective effects of hepatic macrophages. Additionally, suppression of NF-κB by sulfasalazine induces apoptosis of HSC in humans and rats (49). Furthermore, the depletion of macrophages by clodronate liposome reduced IL-1β and TNF-α mRNA in the fibrotic liver (48). Recent research has shown that mitochondrial DNA from hepatocytes of high-fat diet (HFD)-fed mice activates KCs and induces steatosis and inflammation via the stimulator of IFN genes (STING) pathway (50) (Figure 1B). In a mouse model of NASH, fibrosis, inflammation, and steatosis were diminished in the livers of STING-deficient mice (50). The STING agonist, dimethylxanthenone-4-acetic acid, augmented the TNF-α and IL-6 produced by KCs from WT mice, and this increase was attenuated in STING-deficient mice (50). The current literature suggests that activated hepatic macrophages promote the progression of NASH. In contrast, Du et al. found that the expression of TIM-3 on hepatic macrophages is dramatically increased in a methionine- and choline-deficient diet (MCD)-induced NASH model (51). In the same study, TIM-3 deficiency increased the release of ROS by hepatic macrophages and promoted MCD-induced liver fibrosis, as well as steatosis (51) (Figure 1B). These results suggest a mechanism by which hepatic macrophages can inhibit NASH development.

## Recruited Macrophages: Friend or Foe in Nash Progression? (51)

Odegaard et al. demonstrated that, in lethally irradiated mice, an HFD promotes the recruitment of bone-marrow-derived monocytes to the liver; these cells then differentiate into anti-inflammatory macrophages, which provide a protective effect against diet-induced insulin resistance via the peroxisome proliferator-activated receptor δ (PPARδ) pathway (52) (Figure 1B). The adoptive transfer of PPARδ-/- bone marrow into WT mice failed to activate alternative macrophages or attenuate the induced glucose intolerance caused by the HFD (52). In agreement with these finds, Oliver et al. demonstrated that in an overdose of acetaminophen-induced acute liver damage model, high-fructose, high-fat, and high-cholesterol (FFC)-diet-fed mice shows attenuated liver injury than normal-diet-fed mice (53). In the same model, adopting bone-marrow-derived macrophages (BMDMs) from normal-diet-fed mice into FFC-diet-fed mice increases liver damage (53). Single-cell RNA sequencing reveals that these BMDMs from FFC-diet-fed mice downregulate *S100a8/S100a9*, genes encoding inflammatory marker calprotectin, compared with normal-diet-fed mice (53). Additionally, FFC diet also suppresses the TLR4-dependent inflammatory capacity of BMDMs in the mouse NASH model (53). BMDMs from FFC-diet-fed mice are insensitive to LPS stimulation, reflected by less IL-6 and TNF-α production compared with their normal-diet-fed counterparts (53). In contrast, growing evidence has demonstrated that NASH niche favors pro-inflammatory macrophage/monocyte infiltration, and these infiltrated cells increase liver damage and inflammation (54). The fatty acid palmitate can stimulate death receptor 5 on hepatocytes, resulting in release of extracellular vehicles (EVs) (54). The EVs released from lipotoxic hepatocytes have been shown to promote BMDMs toward the pro-inflammatory phenotype characterized by increasing expression of *Il1b* and *Il6* mRNAs (54). Moreover, hepatocyte-lipotoxicity-induced EVs are enriched with integrin α_9_β_1_ (55) and/or CXCL10 (56), which augment pro-inflammatory macrophage infiltration and enhance hepatic fibrosis (Figure 1B). Integrin α_9_β_1_ is required for monocytes to attach liver sinusoidal endothelial; blockade of this interaction by anti-integrin α_9_β_1_ antibody decreases FFC-diet-induced liver fibrosis and injury in NASH mice (55). During hepatic injury, pro-inflammatory macrophages/monocytes are attracted to liver via the CXCL10–CXCR3 axis (57). Compared with those in WT mice, FFC-diet-induced liver injury and inflammation are alleviated in CXCL10–/– mice (56). In a randomized trial, targeting pro-inflammatory monocytes/macrophages by cenicriviroc, a dual antagonist of CCR2 and CCR5, improves hepatic fibrosis in NASH patients (58). One crucial signal that controls the fate of these monocyte-derived macrophages is the type of fatty acids to which the macrophage is exposed. Exposure by saturated fatty acid causes hepatocyte lipotoxicity that then promotes pro-inflammatory macrophage differentiation, whereas stimulation by unsaturated fatty acids activates PPARδ to enhance anti-inflammatory differentiation in NASH (Figure 1B) (52, 59). Taken together, monocytes/macrophages are recruited to the liver during NASH; in response to different compositions of fatty acids, these cells can be differentiated into tissue damage pro-inflammatory macrophages and/or tissue repair anti-inflammatory macrophages; the ratio of two macrophage subsets may determine the role of hepatic macrophage in the pathogenesis of NASH.

## The Role of Hepatic Macrophages in Viral Hepatitis

**Figure 2:**
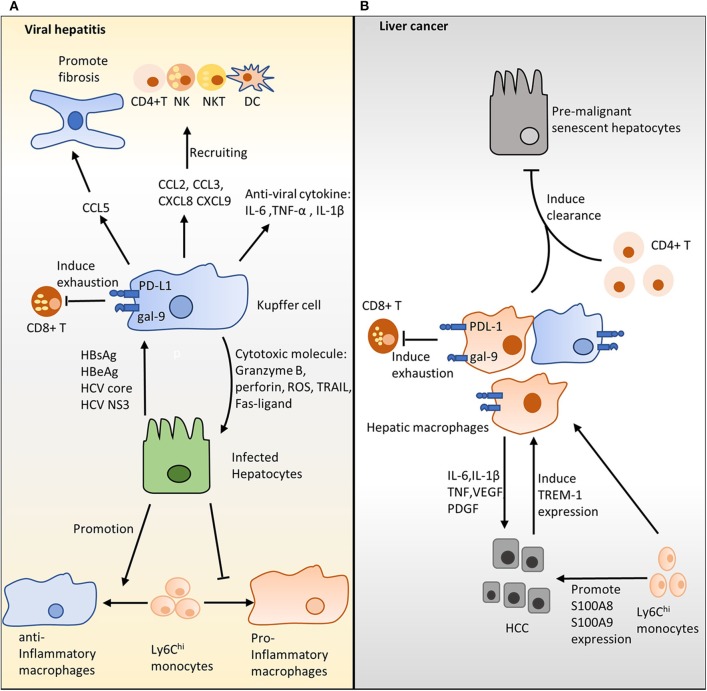
The role of hepatic macrophages in viral hepatitis and hepatocellular carcinoma (HCC). **(A)** Hepatic macrophages and hepatitis B virus (HBV)/hepatitis C virus (HCV). Interleukin (IL)-6, tumor necrosis factor (TNF)-α, and IL-1β produced by Kupffer cells (KCs) show strong antiviral activities. Additionally, KCs may remove infected hepatocytes by producing cytotoxic molecules, including granzyme B, perforin, reactive oxygen species, TNF-related apoptosis-inducing ligand, and Fas-ligand. KCs produce distinct chemokines, including CC- chemokine ligand (CCL)2, CCL3, CXC-chemokine ligand (CXCL)8, and CXCL9, and, together, these chemokines recruit natural killer cells, natural killer T cells, dendritic cells, and CD4+ T cells to infected sites and enhance infection clearance. HCV stimulation induces hepatic macrophages to generate CCL5, which in turn activates hepatic stellate cells and eventually triggers live inflammation and fibrosis. KCs mediate T-cell dysfunction via PD-1/PD-L1 and TIM-3/galectin-9 pathways. Increased HBV inoculum suppresses polarization of pro-inflammation macrophages. **(B)** Hepatic macrophages contribute to HCC. Hepatic macrophages produce IL-6, IL-1β, TNF, vascular endothelial growth factor, and platelet-derived growth factor to promote tumor growth and angiogenesis during HCC. KCs suppress antitumor activity by inducing T-cell dysfunction through PD-L1/PD-1 and galectin-9/TIM-3 in the HCC setting. In contrast, hepatic macrophages assist CD4+ T cells in removing the premalignant senescent hepatocytes that enhance HCC progression. Ly6C^hi^ monocytes increase the expression of S100A8 and S100A9 on cancer cells and promote tumor migration and invasion.

The role of hepatic macrophages in the progression of viral hepatitis is still controversial. Activated KCs, characterized by the upregulation of CD33 and CD163, accumulate in the portal tract during chronic HBV/HCV infection, highlighting the importance of these cells in fighting viral hepatitis (60, 61). KCs are the primary source of IL-1β, TNF-α, and IL-6; these inflammatory cytokines exhibit strong antiviral activity during an infection (62) (Figure 2A). Additionally, it has been shown that KCs may eliminate infected hepatocytes by releasing cytotoxic molecules, such as granzyme B, perforin, ROS, TRAIL, and Fas ligand (63, 64) (Figure 2A). Furthermore, the supernatant from differentiated pro-inflammatory macrophages contains reasonable amounts of IL-1β and IL-6, which inhibit the progression of HBV by decreasing levels of hepatitis B surface antigen (HBsAg) and hepatitis B early antigen (HBeAg) (65).

Several studies have indicated that, in humans, HBV/HCV can directly stimulate hepatic macrophages to trigger inflammatory cytokine secretion, thereby enhancing antiviral activity (15, 66) (Figure 2A). *In vitro* stimulation with HBsAg and HBeAg promoted primary human non-parenchymal liver cells to produce IL-6, IL-8, TNF-α, and IL-1β via the NF-κB pathway (67, 68). Similarly, culturing with HCV enhanced the production of IL-1β and IL-18 by KCs and monocyte-derived macrophages (69, 70). It has been documented that HCV core proteins and nonstructural protein 3 trigger monocyte-derived macrophage activation via TLR1, TLR2, and TLR6 signaling (71). In agreement with these findings, immunofluorescence analysis showed that IL-1β and CD68 are co-localized in liver tissues of chronic HCV patients (72). Apart from inflammatory cytokines, activated KCs also produce CCL2 (73), CCL3 (74), CXCL8 (67), and CXCL9 (74, 75). Together, these chemokines recruit natural killer (NK), NKT, dendritic cells (DC), and CD4+ T cells to infected sites to accelerate infection clearance (74, 75). Although uptake of HBV/HCV by KCs *ex vivo* has not been reported, accumulating evidence from *in vitro* experiments suggests that KCs are involved in HBV/HCV clearance via producing inflammatory cytokines and activating other immune cells.

In contrast, it has been shown that hepatic macrophages are involved in the development of HBV/HCV-induced fibrosis. Incubation with HBV significantly enhanced the generation of the pro-fibrotic growth factor TGF-β1 by primary rat KCs (76). Sasaki et al. found that HCV stimulation induced hepatic macrophages to produce CCL5, which in turn activated HSCs and triggered live inflammation as well as fibrosis (77) (Figure 2A). In the same study, neutralizing CCL5 with an antibody suppressed HSC activation (77). Furthermore, stimulation with the HCV core protein induces programmed death ligand 1 (PD-L1) expression by KCs (78). Similarly, high galectin-9 expression is seen on the KCs of patients with chronic HBV infections (79). Activation of the programmed cell death protein 1 (PD-1)/PD-L1 and TIM-3/galectin-9 pathways in T cells evokes T-cell dysfunction and, thereby, favors the establishment of a chronic infection (78, 79) (Figure 2A).

One hypothesis for these phenomena is that the phenotype of the hepatic macrophages may be shaped by HBV/HCV as the infection progresses. During the early phase of infection, hepatic macrophages are dominated by pro-inflammatory subsets that inhibit virus development by producing cytokines with antiviral activity. In contrast, the chronic hepatitis infection environment suppresses hepatic macrophages polarizing toward the pro-inflammatory phenotype and pushes cells toward the immunoregulation phenotype. Thus, hepatic macrophages show weak antiviral and strong pathological activities in the chronic hepatitis (14). This finding is supported by a recent study showing that an increase in the HBV inoculum attenuated the polarization of monocytes into pro-inflammatory macrophages, evidenced by decreased IL-6 production (65) (Figure 2A). In the same study, exposure to the HBV virus enhanced monocyte anti-inflammatory differentiation, evidenced by increased IL-10 production (65) (Figure 2A). It is likely that a high virus titer suppresses the antiviral activity of hepatic macrophages and polarizes hepatic macrophages toward a tolerogenic phenotype. In agreement with this hypothesis, Faure-Dupuy et al. demonstrated that exposure to HBV attenuated cytokine release by pro-inflammatory hepatic macrophages and enhanced cytokine production by anti-inflammatory hepatic macrophages (65). This modulation suppresses the antiviral surveillance and favors the establishment of an infection (65). Taken together, a high HBV/HCV titer not only inhibits pro-inflammatory macrophage polarization but also promotes macrophages differentiating toward a tolerogenic phenotype, which favors HBV/HCV development by releasing immunoregulation cytokine IL-10.

## Hepatic Macrophages and Hcc

Hepatic macrophages play a crucial role in the pathogenesis of HCC, as evidenced by the accumulation of hepatic macrophages in resections of HCC patients (80) and the liver tissue of chemically induced HCC mice (81). The majority of studies suggest that hepatic macrophages are pro-inflammatory and pro-tumorigenic cells, which inhibit antitumor immunity and favor the establishment of tumors (82–84). Having a large population of hepatic macrophages is associated with poor survival in HCC patients (80, 85). During HCC, hepatic macrophages produce the pro-angiogenic factors, TGF-β, vascular endothelial growth factor (VEGF), and platelet-derived growth factor (PDGF), which, together, promote tumor growth (84, 86) (Figure 2B). Additionally, it has been documented that hepatic macrophages release different mediators, including IL-6, IL-1β, CCL2, VEGF A (VEGFA), and TNF, to augment tumor cell proliferation in HCC (83, 86) (Figure 2B). The evidence for liver macrophage inhibition of HCC growth is limited. The most convincing evidence probably comes from a study of 302 HCC patients, which demonstrated that a high number of CD68+ macrophages is associated with better overall survival (87). Moreover, Kang et al. showed that hepatic macrophages assisted CD4+ T cells in cleaning the premalignant senescent hepatocytes that promote HCC development in an animal model (88). Therefore, two clinical studies with similar clinical–pathologic characteristics but varied in the number of patients have led to contradictory results (80, 87). It is possible that different therapeutic strategies, in particular, post-recurrence therapies, may have been used in these studies (80, 87). CD68 was used to identify tumor-associated macrophages (TAMs) (80, 87). It is widely accepted that TAMs form heterogeneous populations; therefore, the TAM subset contributions to tumor growth progression or inhibition remain to be investigated. This may help to further evaluate the discrepancy between these two studies.

Studies have found that KCs suppress antitumor activity by inducing T-cell tolerance and dysfunction in an HCC setting. KCs have been demonstrated to function as incomplete antigen-presenting cells (APCs) to induce T-cell tolerance (89). This idea is further supported by a recent study which showed that human KCs might exhibit a tolerogenic phenotype (12); they accumulate at the peritumoral stroma expressing high levels of PD-L1 (90, 91) and galectin-9 (92), thereby inhibiting the antitumor response by activating PD-L1/PD-1 and galectin-9/TIM-3 signaling in T cells (Figure 2B). Moreover, the triggering receptor expressed on myeloid cells-1 (TREM-1) is an activating receptor that is widely expressed on monocytes, macrophages, and neutrophils (93). Cancer cell stimulation has been shown to directly increase the expression TREM-1 on KCs, which, in turn, promotes KC activation and HCC progression (93, 94) (Figure 2B). In the same study, *Trem1* deficiency diminished IL-1β, IL-6, TNF, CCL2, and CXCL10 release by KCs and suppressed HCC growth (94). Taken together, interaction between T cells and KCs hinders antitumor response by promoting T-cell exhaustion in HCC.

The role of recruited macrophages in HCC development is highlighted by the importance of the CCL2/CCR2 signaling axis, which is crucial for Ly6C^hi^ monocyte recruitment to inflammatory sites (95). It has been suggested that monocyte recruitment during HCC depends on KCs (96), senescent hepatocytes (97), and tumor-associated neutrophils (98). Conditional media from Ly6C^hi^ monocytes increased the expression of S100A8 and S100A9 in cancer cells and promoted tumor migration and invasion in an experimental liver metastasis model (99) (Figure 2B). In a preclinical model of HCC, blocking CCL2/CCR2 signaling with a CCR2 antagonist reduced Ly6C^hi^ monocyte numbers in the peripheral blood and suppressed anti-inflammatory macrophage polarization in the liver, ultimately inhibiting tumor growth (100). Indeed, a large number of studies have shown that the CCL2/CCR2 pathway involves the recruitment of myeloid-derived suppressor cells (MDSCs) during inflammation, and Ly6C^hi^ monocytes have been shown to be the precursor of MDSCs (101, 102); therefore, the antitumor effect triggered by blocking the CCL2/CCR2 pathway may be partially due to MDSC depletion. To sum up, during HCC progression, macrophages and MDSCs are recruited to the liver via the CCL2/CCR2 axis; these cells have been shown to promote tumor proliferation and metastasis.

## Perspective

A tremendous amount of research over the last few decades has revealed that hepatic macrophages play a central role in the pathogenesis of liver disease. Several strategies have been employed to specifically target hepatic macrophages in different liver diseases (Table 1). Notably, CD11b, F4/80, and Ly6C in mice and CD14, HLA-DR, and CD68 in humans have been widely used to identify KCs; however, these markers may be inadequate to distinguish KCs from recruited macrophages. It has been shown that murine KCs express a unique marker, CLEC4F (11). Meanwhile, single-cell RNA-seq analysis showed that KCs are CD68+ Macro+ in healthy humans (12). Therefore, adding these new markers to the conventional hepatic macrophage identification panel should be considered for precise future investigations into the role of liver macrophage subsets in the development of the liver disease. The recently developed mass cytometry Cyto F technique has been used to study hepatic macrophage in liver disease (55); this technique can simultaneously label up to 350 markers on a single cell, therefore providing a powerful platform to investigate in depth the heterogeneity of hepatic macrophages under different liver diseases as well as pharmaceutical intervention conditions.

**Table 1:** Pharmacological agents targeting macrophages in alcoholic liver disease, non-alcoholic steatohepatitis, viral hepatitis, or hepatocellular carcinoma.

**Target**	**Agent**	**Mechanism of action**	**Phase**	**Clinical trial number**
**ALCOHOLIC LIVER DISEASE**				
Gut bacteria	Combined vancomycin and gentamycin and meropenem	Inhibiting macrophage activation by gut bacteria eradication	Ongoing	NCT03157388
**NON-ALCOHOLIC STEATOHEPATITIS**				
Galectin 3	GR-MD-02	Galectin 3 antagonist on macrophages	Phase 2	NCT02462967
CCR2/CCR5	Cenicriviroc	CCR2/CCR5 antagonist (inhibits monocyte/macrophage infiltration)	Phase 2	NCT02217475
PPARα/δ	Elafibranor	Dual PPARα/δ agonist, PPARδ agonist promotes anti-inflammatory differentiation	Phase 3	NCT02704403
**VIRAL HEPATITIS**				
GM-CSF	Entecavir plus GM-CSF	GM-CSF promotes macrophage differentiation	Ongoing	NCT03164889
GM-CSF	Y peginterferon alpha-2b plus GM-CSF	GM-CSF promotes macrophage differentiation	Phase 2	NCT02332473
**HEPATOCELLULAR CARCINOMA**				
CSF1R	Chiauranib	Multi-target inhibitor that suppresses angiogenesis-related kinases, mitosis-related kinase Aurora B, and CSF1R. Blockade of CSF1R decreases the macrophage differentiation.	Phase 1	NCT03245190
CCR2/5	Nivolumab plus CCR2/5 inhibitor	CCR2/CCR5 antagonist (inhibits monocyte/macrophage infiltration)	Phase 2	NCT04123379

*CCR2, CC chemokine receptor 2, CSF1R, colony-stimulating factor 1 receptor, GM-CSF, granulocyte-macrophage colony-stimulating factor*.

During inflammation, circulating monocytes infiltrate the liver and are involved in the progression of various liver diseases. The phenotypes and roles of monocyte-derived hepatic macrophages are highly dependent on local stimuli during liver disease (103). For example, during fibrosis, a novel monocyte-derived TREM2+ CD9+ scar-associated macrophage has been discovered; this population is expanded in cirrhotic livers and exhibited a pro-fibrogenic phenotype (104). The current M1–M2 model has limitations; this concept cannot define all cell phenotypes, especially macrophages during chronic inflammation and chronic infection liver disease (16). A recent study suggested an extension to the M1–M2 model by showing that, other than M1 and M2 macrophages, human macrophages can be polarized into distinct phenotypes in response to various stimuli (103). Therefore, it is important to precisely describe macrophage populations based on their origins, stimuli, and identification markers (105).

Self-renewing peritoneal macrophages have been shown to migrate to the liver in response to sterile injury (23). Additionally, the spleen is thought to be a reservoir for inflammatory monocytes, which infiltrate the liver and differentiate into hepatic macrophages during liver injury (106). These studies suggest that recruited macrophages are a highly heterogeneous population, composed of subsets with different origins and functions (23, 107). Currently, monocyte-derived recruited macrophages are extensively studied; however, the contributions of peritoneal cavity and spleen-derived recruited macrophages to the pathogenesis of distinct liver diseases are obscured and remain to be explored in the future.

## Author Contributions

LD wrote the first draft of the manuscript. YG, XH, and XS contributed to manuscript revision and read and approved the submitted version.

### Conflict of Interest

The authors declare that the research was conducted in the absence of any commercial or financial relationships that could be construed as a potential conflict of interest.
